# Strategies for improving maternal care for ethnic minority women with obstetric anal sphincter injuries in the UK

**DOI:** 10.1186/s12913-025-12441-1

**Published:** 2025-02-25

**Authors:** Olufisayo Olakotan, Vedhapriya Sudhakar, Jennifer Nw Lim, Mina Bhavsar, Farah Siddiqui, Rabina Ayaz, Gillian O’Brady Henry, Thillagavathie Pillay

**Affiliations:** 1 Department of Neonatology, Women and Children’S Directorate, University Hospitals Leicester NHS Trust, Leicester, UK; 2 Faculty of Education, Health and Wellbeing, University of Wolverhampton, Wolverhampton, UK; 3 Leicester, Leicestershire and Rutland Local Maternity and Neonatal Systems, Leicester, UK; 4 Department of Obstetrics, Women and Children’S Directorate, University Hospitals Leicester NHS Trust, Leicester, UK; 5 Leicestershire Partnership NHS Trust, Leicester, UK; 6 Faculty of Science and Engineering, University of Wolverhampton, Wolverhampton, UK; 7 Department of Population Health Sciences, University of Leicester, Leicester, UK; 8 Neonatal Unit, Leicester Royal Infirmary, University Hospitals Leicester, Leicester, LE1 5WW UK

**Keywords:** OASI, Lived experience, Perinatal health inequalities, Ethnic minority groups

## Abstract

**Background:**

Women from minority ethnic groups in the UK have the highest prevalence of obstetric anal sphincter injuries (OASI), including third- and fourth-degree perineal tears sustained during childbirth. Incorporating the voices of mothers at higher risk of OASI is crucial in developing strategies to improve care and well-being.

**Aim:**

To identify strategies perceived as important by women with lived experience of OASI in Leicester, UK, to improve their care and well-being.

**Methodology:**

Women at high risk of and with lived experience of OASI in Leicester, UK, were invited, through our local maternity and neonatal voices partnership, to participate in a virtual focus group discussion (FGDs). Two FGDs were conducted to accommodate participants' availability. The first session included seven women, while the second session included three women. All participants were from underrepresented groups with lived experiences of OASI within the past one to ten years. A discussion guide was used to explore participants’ perceptions and experiences of OASI care, as well as their views on culturally and linguistically sensitive maternal care strategies. The discussion was recorded and transcribed. The data were analysed using the six-step thematic analysis approach by Braun and Clarke.

**Results:**

The participants identified strategies to provide culturally appropriate care for obstetric anal sphincter injuries, including linguistically accessible prenatal resources, comprehensive midwifery training, updated prenatal education, standardized postpartum care, the inclusion of fathers in prenatal education, empathetic care, building trust, and community engagement and education.

**Conclusion:**

This study highlights disparities in maternal healthcare outcomes for women from diverse ethnic backgrounds who experience OASI. Addressing these inequalities requires incorporating the voices of those directly affected to inform culturally sensitive policies and practices in maternal care.

**Supplementary Information:**

The online version contains supplementary material available at 10.1186/s12913-025-12441-1.

## Background

Obstetric anal sphincter injuries (OASI) are among the most severe complications of vaginal delivery, involving third- and fourth-degree perineal tears that affect the anal sphincter and, in some cases, the rectal mucosa [1]. These injuries have profound implications for women's health, impacting their physical, psychological, and social well-being in both the immediate postpartum period and the long term. A study reports that more than 85% of women having a vaginal birth experience some degree of perineal trauma [1], while OASI occurs in about 3 in 100 women, significantly impacting their physical and social well-being in both the immediate postnatal period and the long term [2]. Several risk factors are associated with OASI include the medicalization of childbirth, such as rising rates of induction, augmented labour, and epidural use; instrumental deliveries like forceps or vacuum-assisted births; macrosomia; prolonged labour [3–5]. The absence of manual perineal support and poor communication during the second stage of labour further increase the risk by leading to uncoordinated pushing or excessive force [6]. Additionally, systemic issues like staffing shortages result in inconsistent care and delayed decisions, exacerbating the likelihood of perineal trauma and severe complications during childbirth [7].

Women described psychological challenges related to sexual intimacy as a result of OASI, such as how their sexual drive and desire had vanished after the injury and the problems related to it [8]. They also described experiencing "weird feelings" in their genitals, such as numbness or oversensitivity, leading to either a lack of sensation or excessive sensitivity during intercourse [9]. In addition to sexual dysfunction, many women reported experiencing anal incontinence, which significantly impacted their daily lives [10]. These physical challenges often left women feeling torn between remaining silent to avoid social disgrace and speaking out to seek help, as anal incontinence and other symptoms were stigmatized [10]. This often led to feelings of isolation, with many women not knowing anyone who shared similar experiences. Women also reported significant short- and long-term morbidities associated with OASI, including chronic pain, incontinence, vaginal prolapse, infections, sexual dysfunction, anxiety, and depression [11, 12]. These persistent issues are often dismissed or misdiagnosed by healthcare providers, leading to inadequate treatment and prolonged suffering. Consequently, women face disruptions in daily life, including limited bonding with their infants and strained relationships with partners due to emotional distress and sexual dysfunction [11]. Financial and social challenges further compound their struggles, highlighting the urgent need for comprehensive postpartum care and recognition of their unique challenges [11, 12].

OASI disproportionately affects underrepresented groups, exacerbated by disparities in access to care and support. Many women experienced a lack of accessibility to healthcare services, while some stated that healthcare providers neglected their health issues and attempted to minimize their problems, even when they were officially identified [8, 13]. To mitigate risks and complications associated with OASI, quality improvement or intervention strategies such as the use of care bundles [14–16], perineal massage [17–19], different maternal birthing positions [20], and warm compresses [21, 22] have been adopted. For example, the OASI care bundle, provides relevant information to women during antenatal period, such as manual perineal protection and specific birthing techniques like mediolateral episiotomy [14–16]. Studies report that women consider antenatal perineal massage practice acceptable [17–19]. They believe it assists them, making them feel more in control and positive about their own labour preparation for birth [18]. Kneeling and all-fours positions for giving birth appear to be more closely associated with an intact perineum compared to sitting, squatting, and using a birth stool, although the associations between maternal position and the degree of perineal tear have not been fully established [20]. Others reported that warm compresses may reduce the incidence of third- and fourth-degree perineal tears [21, 22]. In one Australian trial, women considered warm compresses to be highly acceptable, would recommend them to other women, and reported less pain during birth and on days one and two postpartum [21].

While these strategies have yielded some benefits, addressing the unique needs of women from diverse ethnic backgrounds requires more tailored approaches. Engaging women to identify strategies is essential to overcoming disparities in maternal outcomes [23–26]. Existing literature highlights the value of involving service users in shaping healthcare interventions [27–31]. The aim of this study is to identify strategies perceived as important by women with lived experience of OASI in Leicester, UK, to improve their care and well-being. This need arose from data displayed on the perinatal health inequality dashboards at the University Hospital of Leicester (UHL), which highlighted an increase in third-and fourth-degree tears among ethnic minority women in Leicestershire.

## Methods

### Setting

The focus group discussion (FGD) was organized jointly by the Local Maternity and Neonatal System (LMNS) at the Leicester Royal Infirmary (LRI), which is part of the UHL in conjunction with Leicester Mammas [32], a Leicester-based organization that supports mothers and babies with breastfeeding and other needs. LRI, a level 3 care facility, handles approximately 10,000 births annually and serves as the base for the children's hospital, which provides treatment for various childbirth-related conditions.

### Participants

We specifically extended an invitation to women from underrepresented groups with lived experiences of OASI to participate in the FGD, ensuring our recruitment process targeted communities often overlooked in research. Invitations were sent via email through Leicester Mammas, a community organization supporting pregnant women, new mothers, and their families. Acting as an intermediary, the organization shared the invitation with its members, allowing individuals to respond voluntarily after receiving detailed information about the study's purpose, their role, and their right to withdraw at any time without consequences. A total of sixteen women were contacted. Twelve women responded, with seven women participating in the initial FGD, held on August 15, 2023. A second FGD was conducted on April 10, 2024, with three women to accommodate those who were unable to attend the initial session due to scheduling difficulties.

Eligible participants for both FGD needed to meet the following criteria: be female, 18 years or older, have experienced a third-degree tear within the past one to ten years, possess the ability to read, write, and communicate fluently in English, and provide informed consent. Participants were assured that their anonymity would be protected by omitting names and identifying details from transcripts and publications. However, they were informed that complete confidentiality could not be guaranteed due to the group setting. To mitigate this, participants were requested to respect the privacy of others and refrain from sharing details discussed outside the group. The demographic details of these participants are presented in Table 1 below. This study is reported in accordance with the COREQ (consolidated criteria for reporting qualitative research) guideline.

**Table 1:** Demographics of participants

**Participants**	**Age**	**Experienced of third-degree tears**	**Number of such experiences**	**Number of children**	**Ethnicity**	**Language spoken**
**First focus group participants**						
NF	38	Yes	One	Two	Indian	English
RP	36	Yes	One	Two	Pakistani	English
HV	25	Yes	One	One	Indian	English and Gujarati
BP	40	Yes	One	Three	Indian	English and Gujarati
SS	33	Yes	Two	Two	Indian	English and Gujarati
HS	33	Yes	Two	One	Pakistani	English
AD	30	Yes	One	One	Nigerian	English
**Second focus group participants**						
HA	25	Yes	One	One	Indian	English and Gujarati
YK	34	Yes	One	One	Indian	English, Gujarati, and Urdu
SB	55	Yes	Three	Three	Indian	English and Gujarati

#### Reflexivity

To ensure rigor and address potential biases, we incorporated reflexivity throughout the data analysis process. The stakeholders’ experiences shaped the research questions, focusing on understanding and addressing disparities in OASI outcomes among underrepresented groups. While this expertise was important, we actively worked to mitigate biases by incorporating multiple stakeholder perspectives during the analysis phase. Member checks were conducted to validate the themes and ensure they accurately reflected the participants’ perspectives. Themes were then reviewed and redefined to maintain clarity, consistency, and alignment with the study objectives.

### Data collection

The data collection process involved workshops and focus group discussions (FGDs) to explore and identify strategies that women perceive as important for improving maternal care and support. Facilitated by TP (a neonatologist) and FS (an obstetrician and gynaecologist), the workshop began with a presentation on the medical aspects of OASI, its link to ethnicity, and data from health inequality dashboards, providing context for discussions on the high incidence of OASI among underrepresented women in Leicestershire. FGDs followed to gather the views, preferences, and suggestions of underrepresented women regarding strategies to enhance healthcare services, support mechanisms, and information dissemination. Discussions, conducted virtually via Microsoft Teams, lasted two hours and were audio recorded and transcribed by OO, a skilled qualitative researcher. Participants were encouraged to express themselves freely, fostering an open and inclusive dialogue.

### Data analysis

We used the six-step thematic analysis approach by Braun and Clarke, namely, familiarization, generating initial codes, searching for themes, reviewing themes, defining themes and writing up the results [33]. The data from the FGDs were transcribed verbatim to ensure the accuracy and completeness of the participants' contributions. Two researchers (OO, VS) carefully read through the transcripts multiple times to familiarize with the data. Initial coding was performed independently by two researchers to identify common patterns and trends in the data, group similar codes into categories, and merge them into broader themes. The development of themes involved iterative discussions among the stakeholders to review and redefine emerging themes, ensuring they accurately capture recurring ideas, suggestions, or recommendations shared by participants. Finally, the results were written up in a coherent narrative to effectively present the insights and nuances of the data, highlighting strategies to improve the care and wellbeing of mothers.

## Results

A total of ten underrepresented women residing in Leicester, aged 25 to 55, who had experienced third- and fourth-degree tears during childbirth within the past one to ten years and were able to communicate fluently in English, were recruited. Based on their lived experiences with OASI, these women identified key strategies to improve maternal care and well-being. Table 2 below summarizes the findings and proposed strategies.

**Table 2:** Summary of key findings for improving maternal care for women with lived experiences of OASI

Key Themes	Summary of Findings	Key Strategies
Multilingual resources	They suggested the need for multilingual healthcare resources, including both physical and digital platforms, as well as real-time interpretation services during hospital visits	• Provide healthcare materials in multiple languages (e.g., Gujarati, Hindi) • Utilize apps that offer pregnancy information in native languages • Implement real-time interpretation services during critical hospital visits
Comprehensive training in midwifery and empathetic care	Comprehensive training for midwives to identify and appropriately refer OASI complications. The importance of empathetic communication and trust-building from healthcare providers was emphasized	• Train midwives to improve communication skills and offer empathetic care • Include emotional support and trust-building as part of midwifery training
Comprehensive and detailed prenatal resources	They advocated for more detailed and comprehensive prenatal resources, as well as personalized birthing plans to better prepare mothers for various outcomes	• Develop detailed prenatal resources to better prepare women for complications like OASI • Offer personalized birthing plans co-developed with mothers who have lived experience of OASI • Expand prenatal education to cover risks such as third- and fourth-degree tears
Timely postpartum care and follow up	They recommended standardized postpartum care protocols that include timely follow-up appointments and physiotherapy to aid in recovery	• Standardize follow-up care for women with severe perineal tears • Ensure timely access to physiotherapy as part of postpartum recovery • Provide better access to postpartum care and make medical appointments more responsive
Including partners in prenatal education	The women suggested creating educational resources and classes specifically tailored for fathers, to increase their involvement and help them better understand how to support their partners	• Design educational resources and online classes specifically for fathers • Encourage father participation in prenatal and postnatal care discussions • Increase father involvement to improve their understanding and support for mothers
Community engagement and education	The women pointed out the need for greater engagement with their communities to challenge cultural norms and improve maternal care outcomes. They recommended hospitals collaborate with community leaders to develop culturally sensitive outreach programs and educational initiatives	• Collaborate with established community leaders to develop culturally sensitive outreach programs • Address cultural norms and misconceptions around childbirth through community education • Engage with diverse ethnic communities to build trust and improve maternal healthcare access

### Multilingual resources

One participant identified online platforms as their primary or initial source of health information, with Google being the predominant starting point for all participants. This often led them to trusted websites like the NHS or WebMD. As one noted: *I would generally just Google it, so whatever would come up on Google, and if it was, you know, the NHS website pops up on that, then I would just, you know, look at that" (NF).* While some participants and their family members were proficient in English, many preferred receiving information in their native languages due to the difficulty of understanding complex medical terms in English, their second language. One shared an example of her husband, who, despite working in the UK and being proficient in English, preferred healthcare materials in Gujarati: *"He works here and everything, but he has a whole preference of reading in Gujarati here. When I was giving him the leaflets, he was like some of these words I don't understand so I would have to translate it from Google Translate and then tell them to read it. So, it would be a very big thing about language barrier” (HA).*

Participants noted that while some healthcare services offer translated materials, many patients are unaware of them, limiting accessibility. One participant stated: *"Knowing that that kind of leaflets in our language is available that is the main important thing and where to access it… if we don't even know about language leaflets are available then it's difficult to access” (SB).*

In addition to physical resources, digital platforms were also highlighted as a crucial avenue for providing multilingual health information. One shared her positive experience with an app that offers pregnancy-related information in multiple languages, such as Hindi and Gujarati: *I once saw this app that is also in Hindi, Gujarati, and other languages, which I think it will make more sense to have apps like that” (YK).* Participants also emphasized the importance of real-time interpretation services, particularly during critical moments like hospital visits, with one stating: “*While phone-based interpretation services are available in hospitals, they often fail to offer the same level of comfort and clarity as having an interpreter present in person” (HS).*

### Comprehensive and detailed prenatal resources

Participants shared their personal experiences to highlight gaps in the current system, advocating for the development of comprehensive prenatal resources that could better support women and their families before and after childbirth. One participant shared that inadequate prenatal education left her unprepared for childbirth complications, stressing the need for comprehensive information on potential risks, stating*: “I didn’t expect to have a third- or fourth-degree tear because I was never informed about the possibility. The midwife assured me I’d have a natural birth and never mentioned these complications. It would have been helpful to know what could happen so I could prepare (YK)”.*

Another participant highlighted that her knowledge about childbirth and post-delivery complications, such as requiring stitches for OASI repair, came primarily from family and friends rather than healthcare professionals. She emphasized the need for accessible, detailed resources for women who have experienced complications like OASI: *“My understanding of stitches after delivery was based on what my mother went through 36 years ago and what friends told me. It would be so beneficial to have a dedicated birthing guide or plan developed in collaboration with women who’ve experienced this themselves (BP).”*

They discussed the need for healthcare services to move beyond basic educational materials, advocating for clearer and more detailed guidelines that address childbirth challenges. One explained that the standard information provided during her virtual consultations didn’t cover the complexities of her situation. She suggested that a personalized birthing plans, could significantly improve care: “*When I went for my appointment, I had a virtual consultation with videos and forms. They mentioned risks, but the information wasn’t detailed enough to address the actual challenges I faced. Producing a more detailed birthing guide with healthcare providers would have been incredibly helpful (SS).”*

### Timely postpartum care and follow up

Many participants noted challenges in securing timely medical appointments, both during pregnancy and after childbirth, often leading to delayed postpartum recovery. One participant reflected on her experience, highlighting the broader need for healthcare services to be more accessible and responsive: *"I would only see my midwife every two weeks, and any complications I had, I had to wait to address them. By the time I saw her, it was too late. That was a barrier (YK)."*

Recognizing these gaps, they shared their experiences of advocating for their own postpartum care, particularly in cases of severe tears, highlighting the need for better access to postpartum treatments such as physiotherapy. One participant stated: *"I had to go to A&E to speak for myself before I could get examined. The doctor checked my healing and advised me on my symptoms, but I still couldn’t control my urine. This issue continues to affect me"(SB).* Another participant recounted having to advocate for her own physiotherapy: *"I had to request my own physiotherapy. Not everyone can do that, though. Language barriers or a lack of confidence can prevent many women from seeking the care they need. If I hadn’t been persistent or assertive, it would have taken much longer to get help" (HS).*

Similar concerns were echoed by one participant, who said: *"I remember having to push for more attention after birth because things didn’t feel right. I had to ask for appointments and follow-ups, which shouldn’t be the case when you’ve just had a baby"* (HA). These experiences led participants to question the adequacy of the healthcare system, particularly the lack of standardized follow-up care. One asked*, "Shouldn't there be a standard follow-up for someone who's had a significant tear during childbirth?" (AD).*

### Including partners in prenatal education

The participants highlighted the importance of providing partners with adequate information about OASI and its implications to help them better support their partners. One noted a gap in partners involvement in prenatal education, sharing, *"In a survey, about half of the women said their partners weren't involved in prenatal education or hadn't had a chance to discuss the birth plan" (BP).* Another pointed out that while her partner was generally supportive, his lack of understanding about her OASI experience limited his involvement. She expressed a desire for partners to be better informed about such complications*: "It would be nice for him to know what we go through… So that it would be nice for them to have information. Okay right? She's going through this. What can we do to support her and things like that, so they have information within that?" (SB).*

Participants also suggested that tailored educational resources could help partners become more engaged in understanding OASI and its effects. One participant proposed online classes and informational leaflets to make such knowledge more accessible to partners: *"Maybe even like an online class for dads, new dads, things like that. Like even like a leaflet, right? This is what you can do… I think my husband would be like, right? I would go on it but it was mostly ladies. So, I rather have one where it's just like new-time dads so they can support us while you're going through all that stuff as well" (SS).*

Another participant stated that including partners in antenatal education may enhance their understanding of complications such as OASI, enabling them to better support their partners during labour and postpartum recovery: *"I agree that partners should be more involved in the antenatal education. My husband found our experience profoundly difficult, almost traumatic. In fact, when considering a third child, he hesitated because of the labour experience, not because of the responsibilities that come with a child” (HV).*

### Comprehensive training in midwifery and empathetic care

Participants stated that midwives should be both skilled in managing the birthing process and potential complications and empathetic in their interactions to build trust and provide reassurance. They highlighted the importance of having experienced midwives present during delivery to effectively manage complications and minimize risks, with one stating: "*Midwives need to know how to handle these issues and provide full support to ensure we experience fewer third-degree tears or other complications during pregnancy" (HV).* Another participant shared her frustration with not being taken seriously by midwives, stressing the need for greater training in listening and communication skills. She explained, "*It's crucial for midwives to undergo proper training, especially in listening to the concerns of expecting mothers. Many times, we felt our baby was coming, but we weren't given the attention we needed" (RP).*

Equally important was the role of empathy in fostering trust between mothers and midwives. Participants expressed that genuine care, and emotional support significantly improved their overall experience, especially in critical moments during delivery. One participant described how a lack of empathy in communication led to a breakdown in trust, saying*, "If you approach a mother with genuine care and empathy, she's more likely to listen and trust your advice. I understand the procedure was probably best for my child, but after that, I felt I lost trust in the midwife. I felt apprehensive going forward" (AD).*

Another participant highlighted how differences in midwives’ attitudes affected her comfort during labour. She contrasted two experiences: "*In contrast, my first midwife wasn't someone I clicked with. Her advice didn't resonate with me, and I felt she didn't understand how to help me best. It was only when we had a midwife switch midway through my delivery that I felt more relaxed. The second midwife was calmer and more patient, making me feel like I was in good hands" (RP).*

### Community engagement and education

Participants emphasized that childbirth experiences, particularly those involving complications such as OASI, have lasting physical and emotional impacts on mothers; thus, stating the need for healthcare providers to actively engage with communities to bridge cultural and informational gaps. One cited example from communities, such as the Asian populace, where C-section births are sometimes perceived as taboo. She emphasized that hospitals should be more proactive in addressing these cultural norms by engaging with these communities. *"In some communities, like the Asian one, there's a prevalent discourse about natural vs. C-section births. Breaking these deep-seated norms requires community effort. It's about bridging the knowledge gap and engaging with women from these communities to understand their unique circumstances. Hospitals should be more proactive" (HV)*.

Another participant added that simply reaching out is not enough; it's crucial to collaborate with established or prominent community members. She shared an example of working with the underrepresented groups within the Somali community, which helped increase visibility and trust. "*Simply reaching out might not always be effective. When we approached the Somali community, it was far more impactful to liaise with established channels within the community. We engaged with prominent community figures, like ethnic minority groups from Somali, who trusted us. This automatically increased our credibility, and the community became more receptive" (HS)*.

They also recognized that education needs to be directed both inwardly toward healthcare professionals and mainstream society and outwardly to ethnic communities. One commented on the need for widespread educational efforts: *"We need to make sure that, rather than just reaching out to others, we also educate our own communities… There’s a gap in understanding how culture impacts childbirth choices. We need to address that in a meaningful way” (YK*). Emphasis was placed on the importance of educating communities during outreach efforts, highlighting that learning and growth are essential, with one participant stating: *"We need to ensure that, rather than just reaching out to others from different communities, we educate our own communities as well. Even though it's challenging to teach someone compassion for a culture they don't understand, it's necessary" (RP).*

## Discussion

Our study aimed to identify strategies perceived as important by women with lived experiences of OASI in Leicester, UK, to improve maternal care and well-being. The study highlights the need for clear, concise, and multilingual resources that specifically address the risks, prevention, and management of OASI. Participants emphasised comprehensive prenatal education to better prepare women and their families for potential childbirth complications, as well as timely postpartum care to support recovery and well-being after OASI. Additional priorities included involving partners in antenatal education to enhance support systems, improving midwifery training to foster empathy and communication, and engaging communities to address cultural and informational gaps.

A trusting relationship between the woman and her midwife emerged as paramount for timely detection, effective management, and emotional support following an OASI. Previous studies have underscored the broader significance of such relationships in midwifery care [34–36], but our findings reinforce its specific relevance for OASI. High patient loads, shift work, and time constraints can hinder midwives’ ability to provide meticulous intrapartum monitoring, early recognition of potential risk factors, and consistent follow-up for women with OASI [37]. A partnership case loading model may help reduce some of these barriers by facilitating continuity of care and fostering the deeper connection required to detect and manage OASI effectively [38]. This person-centred approach can further support birth planning, informed consent, and compassionate communication that addresses the woman’s priorities and concerns [39].

The need for up-to-date education and ongoing professional development for midwives is crucial due to the significant impact of midwifery care on maternal and neonatal outcomes [40, 41]. A study underscores the importance of educational interventions, including lectures on anatomy, diagnostics, and suturing skills, enhance midwives' competence in preventing OASIS [42]. The Royal College of Obstetricians and Gynaecologist (RCOG) further emphasises structured training with targeted skill development, regular simulations, and standardised protocols to improve outcomes and reduce missed or mismanaged OASI cases [43]. Additionally, training should address effective communication during the second stage of labour, as misunderstandings between the midwife and the birthing woman highlight the importance of clear, collaborative interaction [42, 44]. Such strategies also help midwives cope with traumatic incidents, fostering greater compassion for themselves and the women in their care.

Involvement of partners also emerged as beneficial, extending beyond general labour support to OASI-specific concerns. While research frequently highlights the importance of a birth companion in improving overall maternity outcomes [45], studies suggest that when partners receive antenatal education, they are more capable of providing practical and emotional support during labour and postpartum recovery [46–50]. This heightened awareness could prove critical in cases of OASI, where consistent and compassionate assistance may speed recovery, bolster women’s confidence in their care, and reduce the long-term physical and psychological repercussions of the injury.

Community engagement may support OASI prevention and management by fostering trust, improving access to antenatal care, and enabling culturally sensitive programme design [51, 52]. A study reported that women appreciated having information about OASI during antenatal care, as it helped them make informed choices [52]. The study also highlighted that enhanced community-level engagement and education could help reduce fear and improve birth experiences. Furthermore, involving relevant stakeholders during community engagement ensure that information and services are tailored to meet the needs of diverse populations, promoting equitable and culturally sensitive care for all women at risk of OASI [52–54].

The findings from the study are currently being used to inform midwives, doctors, and patient groups, contributing to the development of practical outputs aimed at reducing disparities in OASI. Additionally, the results will be disseminated through targeted workshops, professional training sessions, and community outreach programmes to ensure that the findings directly influence clinical practices and policies relating to OASI care.

### Implication for future research

Future research should prioritise addressing structural barriers that limit marginalised ethnic groups’ participation in healthcare decision-making. This involves exploring culturally tailored models to better engage diverse populations and evaluating the long-term effectiveness of strategies designed to improve maternal outcomes, particularly for ethnic minority women. Additionally, research should investigate how culturally sensitive approaches can be integrated into midwifery and healthcare training programmes to improve communication, empathy, and trust-building skills. Such integration could enhance care delivery and foster stronger relationships between healthcare providers and patients.

### Study strength and limitation

The study involved ten participants from Leicester, UK, which reflects a small sample size and limits the generalizability of study findings. A more diverse population may have provided richer and more comprehensive insights. Recruitment was conducted through Leicester Mammas, a local organization, which could have introduced selection bias. Using a more randomized or stratified sampling approach might have enhanced the credibility and representativeness of the sample. Furthermore, excluding participants with limited English proficiency may have affected the dependability of the findings by skewing the perspectives represented.

To enhance the trustworthiness of the findings, member checking was employed, ensuring that the themes accurately reflected the participants’ perspectives. However, beginning the FGD with a presentation on the link between ethnicity and OASI to provide context and stimulate discussion may have influenced participants' responses by framing the discussion around this specific issue, potentially narrowing the focus.

## Conclusion

The study highlights the critical need for maternal care strategies that address the specific needs of women from underrepresented groups who have experienced OASI. By identifying and incorporating the lived experiences of affected women, healthcare systems can develop culturally and linguistically sensitive resources, enhance midwifery training, and standardise postpartum care. Emphasising the involvement of partners in prenatal education and fostering empathetic communication between healthcare providers and patients are essential to improving maternal outcomes. These findings provide valuable insights that can inform the development of culturally sensitive policies and practices in maternal care.

## Supplementary Information

Supplementary Material 1

## Data Availability

The datasets generated and analysed during the current study are not publicly available due to confidentiality but are available from the corresponding author on reasonable request and pending ethics approval from UHL, NHS trust.

## Abbreviations

FGD: Focus Group Discussion
LMNS: Local Maternity and Neonatal System
LRI: Leicester Royal Infirmary
NHS: National Health Service
OASI: Obstetric anal sphincter injury
UHL: University Hospitals of Leicester
