# Supplementary material for: Strategies for improving maternal care for ethnic minority women with obstetric anal sphincter injuries in the UK
Source: BMC Health Serv Res. 2025 Feb 25;25:313. doi: 10.1186/s12913-025-12441-1 (PMC11863463; doi:10.1186/s12913-025-12441-1)
Supplement: Supplementary file 1 [file 12913_2025_12441_MOESM1_ESM.odt]

Focus Group Discussion Guide

Study Title: Severe Perineal Tears during Childbirth amongst ethnic minority women Leicester  

Date: 07/06/23

Moderator: Prof Tilly Pillay and Farah Siddiqui

Introduction

- The moderators introduce themselves, then welcome the participants and explain the objectives of the workshop. 

- Explanation of the basic concepts related to perinatal health, focusing on third degree tears.

- Presentation of the current data and research findings that shows the disparities in the prevalence of third-degree tears among ethnic minorities and white women.

Ground Rules

Encourage honest and open dialogue: There are no right or wrong answers.

Respect: Allow each person to speak without interruption.

Consent: Confirm that everyone is comfortable with the discussion being recorded for research purposes.

Participants introductions 

To help us better understand your unique perspectives, we would like to start by getting to know each of you a bit better. Please introduce yourself by sharing your name, a brief background about yourself, and your experience with childbirth.

FCD question’s 

Section 1: Understanding Current Challenges

- Can you share your experience of the care you received for OASI during and after childbirth?

- What aspects of your care did you find most helpful, and where did you feel there were gaps or unmet needs?

- How do you feel your cultural or ethnic background influenced the care you received, if at all?

Section 2: Identifying Key Needs for Improvement

- What do you think would have made your experience better during pregnancy, childbirth, or postpartum care in relation to OASI?

- Were there any cultural or language barriers that made it difficult for you to access or understand the care being provided?

- What kind of support did you receive from healthcare providers during your experience with OASI, and what more do you think could have been done?

Section 3: Culturally Appropriate Strategies

- In your opinion, what are some specific ways that OASI care can be improved for women from diverse ethnic backgrounds?

- How do you think healthcare professionals, such as midwives or doctors, can better understand and address the unique needs of women from your community?

- Do you feel that more education and involvement of partners or family members during prenatal care would be helpful? If so, in what ways?

Section 4: Specific Strategy Discussions

- What kind of prenatal education or information do you think would help women better understand the risks of OASI and how to prevent it?

- What kind of follow-up care or support would have made your postpartum recovery easier and more comfortable?

- Do you think having community-based support groups or resources for women with OASI would help? What form should these resources take (e.g., virtual support groups, community meetings)?

Closing Questions

- Is there anything else that you would like to share about your experience that we haven’t discussed yet?"

- Do you have any final thoughts or suggestions on how we can better support women who face similar challenges?"

Conclusion

- Thank Participants: Thank each participant for their time and for sharing their valuable insights. Express appreciation for their openness and willingness to contribute to this important discussion.

- Next Steps: Inform participants about the next steps in the research process, including how the information gathered will be used and how they can access the study’s findings if they are interested.
